# Multicenter Experience of Hematopoietic Stem Cell Transplantation in WHIM Syndrome

**DOI:** 10.1007/s10875-021-01155-8

**Published:** 2021-10-26

**Authors:** Alexandra Laberko, Ekaterina Deordieva, Gergely Krivan, Vera Goda, Saleh Bhar, Yuta Kawahara, Kanchan Rao, Austen Worth, David H. McDermott, Dmitry Balashov, Alexey Maschan, Anna Shcherbina

**Affiliations:** 1 Department of Immunology, Dmitry Rogachev National Medical Research Center of Pediatric Hematology, Oncology and Immunology, 1, Samory Mashela str, 117997 Moscow, Russia; 2 Department for Pediatric Hematology and Hemopoietic Stem Cell Transplantation, Central Hospital of Southern Pest- National Institute of Hematology and Infectious Diseases, Budapest, Hungary; 3 Department of Pediatric Hematology Oncology and Critical Care Medicine, Pediatric Bone Marrow Transplantation, Texas Children`s Cancer and Hematology Centers, Baylor College of Medicine, Houston, TX USA; 4 Department of Pediatric, Jichi Medical University School of Medicine, Tochigi, Japan; 5 Department of Immunology and Bone Marrow Transplantation, Great Ormond Street Hospital for Children NHS Foundation Trust, London, UK; 6 Laboratory of Molecular Immunology, National Institute of Allergy and Infectious Diseases, National Institutes of Health, Bethesda, MD USA; 7 Department of Hematopoietic Stem Cell Transplantation, Dmitry Rogachev National Medical Research Center of Pediatric Hematology, Oncology and Immunology, Moscow, Russia

**Keywords:** WHIM syndrome, primary immunodeficiency, congenital neutropenia, human papillomavirus, warts, hematopoietic stem cell transplantation

## Abstract

**Purpose:**

WHIM (warts, hypogammaglobulinemia, infections, and myelokathexis) syndrome is a rare disease, caused by *CXCR4* gene mutations, which incorporates features of combined immunodeficiency, congenital neutropenia, and a predisposition to human papillomavirus infection. Established conventional treatment for WHIM syndrome does not fully prevent infectious complications in these patients. Only single case reports of hematopoietic stem cell transplantation (HSCT) efficacy in WHIM have been published.

**Methods:**

To summarize current information on HSCT efficacy in disease treatment, seven pediatric patients with WHIM syndrome who underwent allogeneic HSCT were identified in five centers worldwide.

**Results:**

All patients presented early after birth with neutropenia. Two of seven patients exhibited severe disease complications: poorly controlled autoimmunity (arthritis and anemia) in one and progressive myelofibrosis with recurrent infections in the other. The remaining patients received HSCT to correct milder disease symptoms (recurrent respiratory infections, progressing thrombocytopenia) and/or to preclude severe disease course in older age. All seven patients engrafted but one developed graft rejection and died of infectious complications after third HSCT. Three other patients experienced severe viral infections after HSCT (including post-transplant lymphoproliferative disease in one) which completely resolved with therapy. At last follow-up (median 6.7 years), all six surviving patients were alive with full donor chimerism. One patient 1.4 years after HSCT had moderate thrombocytopenia and delayed immune recovery; the others had adequate immune recovery and were free of prior disease symptoms.

**Conclusion:**

HSCT in WHIM syndrome corrects neutropenia and immunodeficiency, and leads to resolution of autoimmunity and recurrent infections, including warts.

## Introduction

Warts, hypogammaglobulinemia, infections, and myelokathexis (WHIM) syndrome is a rare autosomal dominant primary immunodeficiency (PID), caused by *CXCR4* gene mutations [1]. CXCR4 is a chemokine receptor expressed by hematopoietic stem and progenitor cells, mature leukocytes, and some non-hematopoietic cells [2]. *CXCR4* gene gain of function mutations in WHIM syndrome lead to a phenotype of combined immunodeficiency and abundant mature apoptotic neutrophil accumulation in bone marrow, or myelokathexis, resulting in neutropenia in peripheral blood [3]. Although the acronym WHIM is formed by classic disease symptoms, a recent review of WHIM syndrome patients by Heusinkveld et al. found myelokathexis to be the only pathognomic sign detectable in all patients [4]. Other clinical presentations vary and include bacterial upper and lower respiratory tract and skin infections, human papillomavirus (HPV) positive warts, as well as laboratory findings such as neutropenia, lymphopenia, hypogammaglobulinemia, and thrombocytopenia. Despite the features of combined primary immunodeficiency (PID), opportunistic infections other than HPV are not common in WHIM syndrome. Up to 18% of patients develop autoimmunity (Geier CB et al, submitted), and up to 30% develop lymphoid and HPV-related malignancies at an older age (19–65 years) [5, 6].

While hematopoietic stem cell transplantation (HSCT) is a well-established option to cure a variety of PID [7], only a few single case reports reporting a curative effect of HSCT in WHIM syndrome have been published so far [8–10].

The current study describes a multicenter HSCT experience in a series of patients with WHIM syndrome.

## Patients and Methods

Seven pediatric patients with WHIM syndrome who underwent allogeneic HSCT were identified in five centers worldwide (Table 1) and retrospectively included in the current study. The details of disease presentation in P4 [11], HSCT information and outcomes of P3, P4, and P5 [8, 9, 12, 13] were previously published.

**Table 1:** Patients’ characteristics

Patient #	Center*	Sex	Disease onset	*CXCR4* mutation**	Age at *CXCR4* mutation detection	*CXCR4* mutation inheritance	Disease symptoms							
							Neutropenia/myelokathexis	Thrombocytopenia	Gingivitis	HPV infections	Oto/sino/pulmonary infections	Other infections	Developmental defects	Other symptoms
P1	Russia	M	at birth	c.1000C>T	7 mon	From affected mother	Yes	Yes	No	No	No	No	Ductus arteriosus, nephromegaly, hydrocele, NDD	Anemia
P2	Russia	M	2 months	c.1027G>T	5 y	De novo	Yes	Yes	No	No	Yes	Acute gastroenteritis	Severe combined heart defect, ectopic left kidney, inferior vena cava duplication, joint hypermobility, NDD	No
P3	Russia	F	4 months	c.970_971insTCCT	4,3 y	De novo	Yes	Yes	No	No	Yes	Furunculosis, CMV viremia	Growth delay, umbilical hernia, talipes valgus	Immune anemia, arthritis
P4	Hungary	F	1 year	c.1013C>G	5,5 y	De novo	Yes	No	No	No	Yes	No	No	No
P5	Japan	F	10 months	c.1000C>T	1 y	De novo	Yes	No	No	No	No	No	No	No
P6	USA	F	at birth	c.969_970insG	2 y	From affected mother	Yes	Yes	Yes	Yes	Yes	Omphalitis, candidal diaper rash, acute gastroenteritis	No	Anemia, reticulin myelofibrosis
P7	UK	F	at birth	c.954delC	7 mon	From affected father	Yes***	No	No	No	Yes	No	No	No

*Dmitry Rogachev National Medical Center of Pediatric Hematology, Oncology and Immunology, Moscow, Russia; Central Hospital of Southern Pest- National Institute of Hematology and Infectious Diseases, Budapest, Hungry; Jichi Medical University School of Medicine, Tochigi, Japan; Texas Children`s Cancer and Hematology Centers, Houston, USA; Great Ormond Street Hospital, London, United Kingdom

**based on CXCR4 sequence given in NM_003467.2 and 003467.3

***no bone marrow investigation to demonstrate myelokathexis was performed

*F* female, *M* male, *HPV* human papilloma virus, *NDD* neurodevelopmental delay

The main characteristics of the patients and details of disease presentation are shown in Table 1. All patients presented early after birth (median age of disease onset 0.85 years) with neutropenia. Myelokathexis in bone marrow was later demonstrated in 6/7 (was not investigated in one). In the course of the disease, 5/7 patients developed thrombocytopenia and 3/7 anemia, presumed to be of immune origin in all but one. In P6, cytopenia (progressive anemia, thrombocytopenia and the need of increasing doses of granulocyte – colony stimulating factor (G-CSF)) was caused by bone marrow reticulin fibrosis. Five patients had lymphopenia with a substantial decrease of both CD3+ and CD19+ cells. Hypogammaglobulinemia was found in 6/7. Patients’ pre-HSCT blood counts, lymphocyte subsets, and immunoglobulins levels are shown in Table 2. Five patients experienced upper and/or lower respiratory tract infections that resolved without sequalae; three patients developed skin and intestinal infections. P6 developed mild cutaneous warts at the age of three years; no HPV infection was observed in the other patients. P3 had rheumatoid arthritis and Coombs-negative autoimmune hemolytic anemia.

**Table 2:** Patients pre- and post- HSCT laboratory parameters

Patient #	HSCT	Age/time after HSCT	Whole blood counts x*10*^*9*^*/l*				Lymphocyte subsets x*10*^*9*^*/l*					Immunoglobulin levels, *g/l*		
			WBC	PMN	PLT**	Lymph	CD3+	CD4+	CD8+	CD19+	CD3-16+56+	IgG	IgM	IgA
P1	Pre	3 months	*3.92*	*0.17*	285 *(44)*	3.61	*1.57*	*1.19*	*0.29*	0.62	1.02	*2.86*	1.11	0.34
	Post	–	–	–	–	–	–	–	–	–	–	–	–	–
P2	Pre	1.9 years	*1.1*	*0.08*	*167 (51)*	*0.87*	*0.88*	*0.74*	*0.06*	*0.23*	0.31	*2.2*	0.8	0.2
	Post	+1.4 years	*2.63*	1.8	*88*	*0.55*	*0.42*	*0.24*	*0.07*	*0.12*	-	6.3	0.87	0.31
P3	Pre	2.7 years	*0.63*	*0.04*	217 *(22)*	*0.49*	*0.38*	*0.26*	*0.09*	*0.03*	0.12	*3.99*	*0.23*	*0.27*
	Post	+5.7 years	7.34	3.44	311	3.05	2.28	*0.84*	1.32	0.53	0.18	10.6	1.5	1.2
P4	Pre	5 years	*2.4*	*0.61*	265	1.92	-	*0.54*	*0.1*	*0.04*	0.11	*4.02*	0.45	0.43
	Post	+12.6 years	6.7	3.9	209	2.26	1.7	0.95	0.57	0.24	0.15	6.7	0.85	0.96
P5	Pre	3 years	*0.5*	*0.01*	189	*0.46*	*0.25*	*0.18*	*0.04*	*0.02*	0.15	*2.7*	0.61	0.48
	Post	+5 years	5	2.2	177	2.45	2.13	0.95	1.03	0.31	-	8.6	0.8	0.62
P6	Pre	3.8 years	*2.93*	*1.16*	*41*	*1.2*	*0.63*	*0.42*	*0.17*	*0.14*	0.15	2.2*	0.99*	0.56*
	Post	+6.5 years	6.6	3.2	258	2.9	1.6	0.92	0.62	0.54	*0.08*	12.1	1.7	0.78
P7	Pre	7 months	*3.6*	*0.1*	394	3.4	2.06	1.63	0.4	*0.15*	*0.01*	5.2	0.9	0.5
	Post	+2 years	6.6	2.2	292	2.22	1.87	1.04	0.78	*0.15*	0.16	7.6	0.71	0.64

*WBC* white blood cells, *PMN* polymorphonuclear leukocytes (neutrophils), *PLT* platelets, *lymph* lymphocytes

As normal ranges for ages varied between different laboratories, abnormal values were highlighted with italic. In all, but P6, immunoglobulin levels were tested before immunoglobulin replacement and G-CSF therapy initiation

*P6 pre-HSCT immunoglobulin levels were tested at the age of 1.8 years

**Pre-HSCT platelet values in P1, P2, and P3 are displayed in parentheses

Six of seven patients were treated with routine intravenous immunoglobulin (IVIG) replacement and 5/7 received G-CSF therapy to correct neutropenia (Table 3); 6/7 patients were on antimicrobial prophylaxis. The median age of IVIG and G-CSF initiation was 1.8 years. P3 received a combination of steroids (maximum dose of methylprednisolone 2 mg/kg/day) and cyclosporin A to control arthritis and autoimmune hemolytic anemia. While on immunosuppressive therapy, P3 developed cytomegalovirus (CMV) viremia and received ganciclovir until HSCT, and foscarnet in the post-HSCT period.

**Table 3:** Hematopoietic stem cell transplantation details

Patient #	Pre-HSCT therapy	Age at therapy initiation, years	Reason for HSCT	Age at HSCT, years	Conditioning regimen	Conditioning type**	GVHD prophylaxis	Donor	HLA match	Stem cell source	Graft manipulation	Graft composition***			Engraftment, days		HSCT outcome
												NC *х10*^*8*^*/kg*	CD34+ *x10*^*6*^*/kg*	CD3+ *x10*^*6*^*/kg*	Neutr	Plt	
P1	IVIG G-CSF 8/d azithromycin fluconazole	0.25	Poor response to high doses of G-CSF, worsening thrombocytopenia	1.4	Treo 42 Flu 150 TT 10 Thymo 5 Rit 100	MAC	No	MUD	10/10	PBSC	TCRαβ+/ CD19+ depletion	7.16	10.53	16.76 (15.75)	+12	+15	Graft rejection d+27, died post 3^rd^ HSCT
P2	IVIG G-CSF 11/d SMP/TMP fluconazole	1.8	Worsening neutropenia and thrombocytopenia	5.8	Treo 42 Flu 150 TT 10 Thymo 5 Rit 100	MAC	No	MMFD	5/10	PBSC	TCRαβ+/ CD19+ depletion	15.4	10.64	18.54 (51.5)	+17	+15	Alive 1.4 years
P3	IVIG G-CSF 20/d SMP/TMP fluconazole CsA, steroids	2.8	Poor control of autoimmunity, worsening neutropenia	3.7	Treo 42 Flu 150 ATGAM 90	RIC	Tacro d+60 Mtx	MUD	10/10	PBSC	TCRαβ+/ CD19+ depletion	8.05	14.0	19.0 (0.8)	+15	+12	Alive 8 years
P4	IVIG G-CSF 5/2d	5.5	Recurrent infections, worsening neutropenia	9.3	Bu 12 Cy 200 Thymo 5	RIC	Mpred d+27 CsA d+384	MSD	12/12	UCB	No	0.36	0.12	0.75	+32	+38	Alive 12.8 years
P5	IVIG	1	Pre-emptively	3.8	Mel 140 Flu 125 TBI 3Gr	RIC	Tacro d+30 CsA d+180 Mtx	MMFD	7/8	BM	No	0.72	3.34	–	+14	+21	Alive 5.7 years
P6	IVIG G-CSF 5-8/d cefuroxime	1.9 1 month	Recurrent infections, myelofibrosis	4.3	Bu* Cy 200 Alem*	MAC	Tacro d+100 Mtx	MUD	10/10	BM	No	7.5	7.2	–	+23	+29	Alive 7.7 years
P7	itraconazole, azithromycin, ciprofloxacin	–	Pre-emptively	2.6	Treo 36 Flu 150 Alem 1	RIC	CsA d+110 MMF d+60	MUD	12/12	PBSC	No	33.7	50	780	+13	+12	Alive 2.7 years

*IVIG* intravenous immunoglobulin, *G-CSF* granulocyte – colony stimulating factor (doses in μg/kg/days), *TMP/SMP* trimethoprim/sulfamethoxazole, *MUD* matched unrelated donor, *MMFD* mismatched family donor, *MSD* matched sibling donor, *UCB* umbilical cord blood, *PBSC* peripheral blood stem cells, *BM* bone marrow, *GVHD* graft-versus-host disease, *MMF* mycophenolate mofetil, *NC* nucleated cells, *Neutr* neutrophil, *Plt* platelet

*Treo* treosulfan, g/m^2^; *TT* thiotepa, mg/kg; *Flu* fludarabine, mg/m^2^; *Cy* cyclophosphamide, mg/kg; *Bu* busulfan, mg/kg; *Mel* melphalan, mg/m^2^; *TBI* total body irradiation; *Thymo* thymoglobulin, mg/kg; *Alem* alemtuzumab, mg/kg; *Rit* rituximab, mg/m^2^; *ATGAM* mg/kg; *CsA* cyclosporin A, *Tacro* tacrolimus, *MMF* mycophenolate mofetil, *Mpred* methylprednisolone, *Mtx* methotrexate 10mg/m^2^ days +1, +3, +6

GVHD prophylaxis is indicated with days of withdrawal. Serotherapy timing was as follows: in P1, P2, P3 days − 5 to − 4, in P4 days − 4 to − 1, in P6 days − 5 to − 2, in P7 days − 8 to − 4

*The total doses of busulfan and alemtuzumab given were 0.8 mg/dose 16 times and 5 mg/dose 4 times (adjusted for 15,1-30 kg body weight), respectively

**according to Shaw et al. definition [17]

*** The numbers of TCRαβ+ cells х10^3^/kg are displayed in CD3+ column in parenthesis

In all patients, the diagnosis of WHIM was confirmed by detection of *CXCR4* gene mutations (Table 1). In P1 and P2, *CXCR4* gene mutations were found by custom-panel sequencing performed using a next-generation sequencing method and subsequently confirmed by Sanger sequencing, while in P3, P4, P5, P6, and P7, only Sanger sequencing was used. All, but P7, mutations were previously investigated functionally; each of them results in a truncated receptor, which was described elsewhere. The mutation in P7 is predicted to result in the truncated protein.

Four patients had *de novo* mutations of the *CXCR4* gene and in three patients, it was inherited from an affected parent. The disease course in affected parents varied: P6’s mother had only warts and recurrent pneumonia, while P1’s mother had debilitating frequent lower respiratory tract and soft tissue infections with extensive warts. P7’s father had frequent lower respiratory tract infections, tuberculosis, and both P6 and P7 parents required regular hospital admissions and/or surgical treatment. *CXCR4* gene mutations were excluded in the family donors of P2 and P4 before HSCT.

For donor chimerism, real-time quantitative polymerase chain reaction (PCR) assessment of the insertion/deletion short tandem repeats or variable number tandem repeats polymorphisms was performed. The presence of more than 95% donor-derived cells in peripheral blood was considered as full donor chimerism.

Neutrophil engraftment was registered on the first of three consecutive days of an absolute neutrophil count above 0.5 × 10^9^/l, and platelet engraftment was registered on the first of three days of platelet counts more than 20 × 10^9^/l without platelet transfusions for seven consecutive days. Loss of donor cells following established engraftment, proven by detection of more than 90% of recipient chimerism in the peripheral blood, was defined as graft failure.

For graft-versus-host disease prophylaxis, TCRαβ+/CD19+ graft depletion was used in P1, P2, and P3. The depletion was performed via Miltenyi Biotec (Bergisch Gladbach, Germany) instructions.

Anti-Epstein-Barr virus (EBV) cytotoxic T-lymphocytes (CTL) used in P7 were generated and stored by the CTL bank held at the University of Aberdeen, UK, in association with the Scottish Blood Transfusion service, according to the described earlier methodology [14].

## Results

In most of the patients, poor disease control (detailed in Table 3) served as indication for HSCT and other patients received HSCT preemptively to prevent the risk of malignancies and to preclude the severe disease course observed in the affected parent. The median age at HSCT was 3.7 years (range 1.4–9.3). All patients described received allogeneic HSCT using different approaches (the details of donors, graft source and composition, conditioning regimens, and post-transplant immunosuppression are shown in Table 3).

The median current follow-up post-HSCT in survivors is 6.7 years (1.4–12.8). All patients engrafted; the median of both neutrophil and platelet engraftment was day + 15 (Table 3). At day +27, P1 developed graft rejection and in five months received a second HSCT from an alternative unrelated donor. Neutrophil and platelet engraftment occurred at day + 17 and + 16, respectively, which was followed by poor graft function and rejection at day + 72. After the second graft rejection, the patient remained neutropenic and lymphopenic and developed poorly controlled multiple infections (adenovirus (ADV) viremia, roto-/noroviral, ADV enterocolitis, *Clostridium difficile* colitis). Due to the absence of alternative curative options, a third allogeneic HSCT from another unrelated donor was performed, followed by non-engraftment and death from disseminated ADV infection at day + 20. All second and third HSCT details are shown in Table 4.

**Table 4:** Second and third HSCT details in P1

# of HSCT	Age at HSCT, years	Donor	HLA match	Conditioning regimen	GVHD prophy-laxis	Stem cell source	Graft manipu-lation	Graft composition*			HSCT outcome
								NC *х10*^*8*^*/kg*	CD34+ *x10*^*6*^*/kg*	CD3+ *x10*^*6*^*/kg*	
2	1,8	MUD #2	9/10	Flu 150 Mel 180 TAI 4 Gr Cy 100 Pler +G-CSF Thymo 5 Rit 100	CsA	PBSC	TCRαβ+/ CD19+ depletion	5,19	9,92	3,12 (16.62)	Neutrophil engraftment d+17. Graft rejection d+72
3	2,2	MUD #3	9/10	Flu 100 Cy 100 Thymo 10	CsA Abat	PBSC	TCRαβ+/ CD19+ depletion	3,07	9,86	4,31 (6.17)	Death d+20

*Flu* fludarabine, mg/m^2^; *Cy* cyclophosphamide, mg/kg; *Mel* melphalan, mg/m^2^; *TAI* thoracoabdominal irradiation; *Thymo* thymoglobulin, mg/kg; *Rit* rituximab, mg/m^2^; *Pler +G-CSF* plerixafor 0,24 mg/kg/d for 3 days + granulocyte – colony stimulating factor 10 μg/kg/d for 5 days; *CsA* cyclosporin A, *Abat* abatacept 10 mg/kg days − 1, + 7, + 14

* The numbers of TCRαβ+ cells × 10^3^/kg are displayed in CD3+ column in parenthesis

P7 at one month post-HSCT developed grade II skin acute graft-versus-host disease (GVHD), which responded well to 1 mg/kg/day steroid therapy. Despite patient and donor being EBV negative pre-transplant, during the steroid tapering, at day + 56, the patient developed EBV viremia with 2,4 million EBV copies per ml of blood, accompanied by ADV viremia which resolved after cidofovir therapy. EBV infection did not respond to rituximab treatment (375 mg/m^2^ given once a week 4 times). At day + 95, while steroids and mycophenolate mofetil had ceased, and cyclosporin A was being weaned (lymphocyte count at the time 0.25–0.45 × 10^9^/l), repeated investigations showed liver lesions, extensive mediastinal and mesenteric lymphadenopathy, and bowel wall thickening. A low level of EBV was detected in the cerebrospinal fluid, with no lesions found in the brain by magnetic resonance imaging. An abdominal mass biopsy revealed CD20+ EBV+ polymorphic post-transplant lymphoproliferative disease (PTLD) of predominantly recipient cell origin, despite full donor chimerism in the peripheral blood (PTLD morphology is shown in Picture 1). The patient received chemotherapy with cyclophosphamide, vincristine, and prednisolone. The treatment was complicated by vancomycin-resistant *Enterococcus* bacteremia, which resolved upon antimicrobial treatment, and posterior reversible encephalopathy. No response to 2 cycles of chemotherapy was seen, with persistent lymphadenopathy, liver lesions, and EBV viremia. Finally, a third-party donor anti-EBV specific CTL infusion was performed, which led to complete resolution of PTLD, confirmed by repeat blood EBV PCR, imaging of abdominal lymphadenopathy and liver lesions, and bone marrow aspirate.

**Picture 1:**
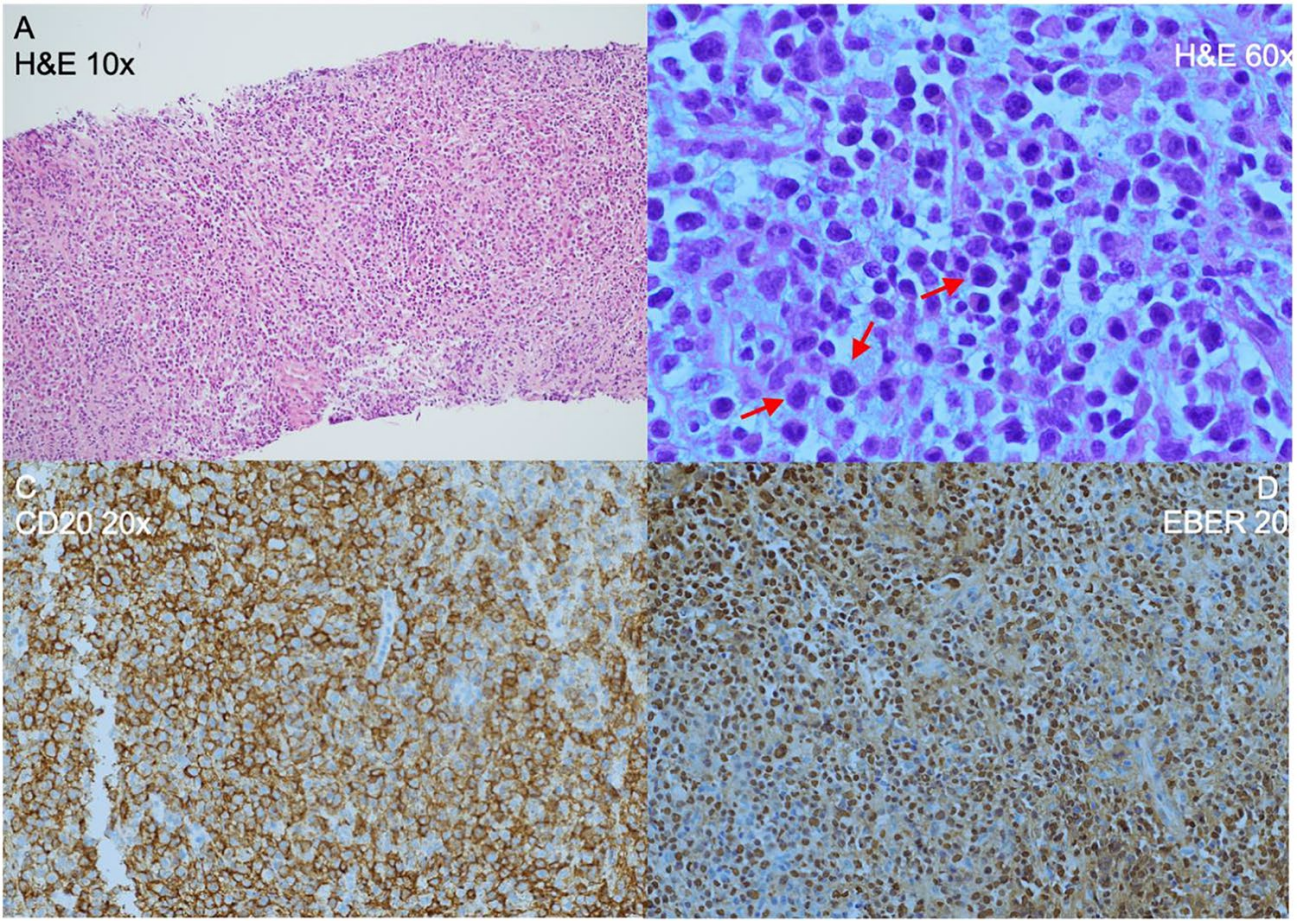
Histology of abdominal lymph node demonstrating EBV driven polymorphic post-transplant lymphoproliferative disease in P7. **A** 10x H&E stain: demonstrates loss any normal lymph node architecture. Lymphoid cells arranged in sheets throughout the sample. **B** 60x H&E stain: many scattered immunoblast like cells (illustrated cells marked), with large nuclei and little cytoplasm. Areas of focal necrosis. **C** 20x CD20 immunostaining: staining demonstrates strong and diffuse CD20 positivity throughout the biopsy. **D** 20x EBER staining. EBV staining is strong and diffuse within lesion cell nuclei. No evidence of MYC or IGH rearrangement was found by fluorescent in situ hybridization

No life-threatening post-HSCT complications were observed in the other patients. P6 had acute grade II skin GVHD, which resolved with steroid therapy. Later, she experienced transient ADV and human herpes virus-7 viremia, mild BK virus cystitis, and *Corynebacterium* bacteremia. Resolution of warts in P6 was observed by 4–6 months post-HSCT with no additional therapy. About a year after HSCT, she developed several molluscum contagiosum lesions, which resolved with liquid nitrogen cryotherapy and an antiviral cream.

At day + 11, P3 developed reactivation of pre-existing CMV viremia, followed by BK virus cystitis. Despite CMV viremia resolution and immunosuppression cessation, after discontinuation of antiviral therapy, at day + 71, CMV viremia recurred and at day + 88, a retinal inflammatory focus was found. Both CMV viremia and retinitis of presumed CMV etiology resolved on antiviral therapy. Except for P6 and P7, no other patients had grade II-IV acute or chronic GVHD.

In the early post-HSCT period, despite full donor chimerism, P2 remained thrombocytopenic with maximum level of platelets 90 × 10^9^/l. At 4.5 months post-HSCT, romiplostim therapy was initiated with good, but short-term response. At 9 months post-HSCT, thrombocytopenia responded well to treatment with several courses of high dose IVIG. However, at last follow-up, 1.4 years after HSCT, the patient still has moderate thrombocytopenia and delayed lymphocyte recovery; however, he is free of infections and does not require IVIG replacement. In the other patients, all baseline hematologic and immune manifestations of WHIM syndrome resolved after HSCT.

All six surviving patients achieved full donor whole blood chimerism after HSCT. At last follow-up, all retained complete donor chimerism and were independent of immunoglobulin replacement. All but P2 had adequate immune recovery and were free of any symptoms of prior disease. Patients’ post-HSCT blood counts, lymphocyte subsets, and immunoglobulin levels are shown in Table 2. P3 had growth delay, P4 immune hypothyroidism and hypogonadism requiring hormonal replacement therapy. No long-term complications in the other patients were observed. In P4 cervical investigation, performed at long-term post HSCT follow-up, revealed no pathologic findings.

## Discussion

WHIM syndrome is a rare congenital disease, which incorporates features of combined immunodeficiency, congenital neutropenia, and predisposition to HPV infection [15]. Conservative therapy with immunoglobulin replacement, G-CSF, and more recently plerixafor does not provide complete control of infectious and autoimmune episodes in WHIM patients [4], (Geier CB et al, submitted) and cannot prevent malignancies [5]. HSCT is a well-established treatment option for many PIDs. As case series of WHIM syndrome are scarce, there are no agreed indications for HSCT specifically determined for this disorder. We retrospectively collected the data of seven pediatric patients with WHIM syndrome, who received HSCT for a variety of reasons and with diverse approaches used.

Of seven reported patients, one died of infectious complications following two graft rejections. Graft failure is relatively common after HSCT in congenital neutropenia [16]. P1 in our study developed graft rejection, despite a myeloablative conditioning regimen [17]. However, most of the patients in the group received reduced intensity conditioning regimens and did not experience severe graft dysfunction, although a potential role of TCRαβ+/ CD19+ graft depletion used in P1 must be also considered in graft rejection [12]. Moreover, P1 developed rejection after the second and third HSCT with more immunosuppressive conditioning regimens, also containing plerixafor. Plerixafor was shown to partially correct neutropenia and lymphopenia in WHIM syndrome [18, 19] and to facilitate stem cell engraftment in some disorders [20, 21]. None of the measures improved the second HSCT outcomes in P1. To evaluate the impact of conditioning regimen type and T-cell elimination methods on graft function in WHIM patients after HSCT, a larger group of patients needs to be studied.

The incidence of EBV-related lymphoid malignancies is increased in WHIM patients [22–24]. None of the patients in our study had malignancies pre-HSCT, which might be due to their young age at the time of HSCT. However, one patient developed EBV-driven polymorphic PTLD of recipient cell origin despite being EBV negative pre-HSCT. Four of seven patients in our study experienced significant viral infections post-HSCT, although in one, severe infections followed graft rejection and in two others infection developed during systemic steroid therapy for GVHD. PID patients, especially with combined immune defects, are known to have higher predisposition to some viral infections after HSCT [13, 25], so further investigation of these risks in WHIM patients is required. Of note, high risk of viral infections in these patients may also argue against more aggressive lymphodepletion, despite the existing risk of graft rejection, observed in one of the patients.

HSCT is known to correct congenital immune and neutrophil defects; however, it does not rescue some patients from a severe post-HSCT HPV infection course [26]. In light of the risks of HPV-related malignancies in WHIM syndrome, the ability of donor-derived immunity to control HPV infections is a crucial question for these patients. Moens et al. reported an exacerbation of HPV infection shortly after HSCT, which was treated with imiquimod and resolved completely 1.5 years post-HSCT [10]. In the current study, the patient who exhibited warts before HSCT demonstrated complete resolution of warts at 4–6 months after HSCT. One patient experienced post-HSCT mild molluscum contagiosum, but none of five surviving patients who had no warts pre-HSCT developed HPV infection after HSCT. Nevertheless, longer follow-up and larger series of patients are needed to estimate the risk of HPV infection and both HPV-related and non-related malignancies in WHIM syndrome after HSCT. Interestingly, although T cells play an important role in anti-HPV immunity [27], a WHIM patient in whom a chromothripsis event corrected defective myelopoiesis, but not lymphopoiesis, cleared HPV infection [28]. This suggests that myeloid cells might play a major role in HPV control in WHIM syndrome. Since all our patients had full donor chimerism at the last follow-up, the level of donor lymphocyte and myeloid lineage chimerism needed to control WHIM syndrome symptoms remains uncertain.

Interestingly, reticulin myelofibrosis observed in P6 was earlier described only in older patients after long-term G-CSF treatment [10, 19]. Severe neutropenia in WHIM syndrome seems to cause less fatal infectious complications, observed in other severe congenital neutropenia, due to neutrophil mobilization from bone marrow to peripheral blood during bacterial infection episodes [5]. However, the risks of myelofibrosis on prolonged G-CSF therapy are of concern. Plerixafor therapy, allowing G-CSF cessation, was demonstrated to ameliorate fibrotic changes in adult patients [19], although plerixafor so far is not widely available for pediatric patients. Notably, P2 a year post-HSCT remains mildly thrombocytopenic and with delayed immune recovery. Due to good response to IVIG therapy, his thrombocytopenia was suspected to be of immune origin, although no bone marrow trephine investigation was performed pre- and post-HSCT to exclude myelofibrosis. Longer follow-up is needed to evaluate platelet and lymphocyte counts in this patient.

Although HSCT in adult patients with longer history of infections and multiple comorbidities might lead to higher risks of post-HSCT complications, Moens et al. reported successful HSCT of an adult patient with WHIM syndrome with a history of refractory warts, recurrent herpes simplex virus infection, and myelofibrosis [10]. Due to improving results of HSCT in PID and better HSCT outcomes in younger patients without poorly controlled disease complications and associated disorders, the modern standard of PID care is to consider HSCT in patients at risk of life-threating complications before their development [7]. In our case series, only two of seven WHIM syndrome patients exhibited severe disease complications: poorly controlled autoimmunity and immunosuppression-related CMV infection in one and progressive myelofibrosis and recurrent infections in another. Other patients received HSCT to correct milder disease symptoms and/or to preclude severe disease course seen, either in parents or reported in the literature.

Based on our data, HSCT in WHIM syndrome patients cannot be considered as a completely safe treatment and risks of life-threating transplant-related complications and death still remain. However, no other treatment options capable of preventing an often devastating disease course are currently available for WHIM. Regular plerixafor therapy does not seem to completely resolve all disease complications [19], while our transplantation experience demonstrates complete resolution of the majority of disease symptoms. Although there is evidence of possible gene therapy efficacy in WHIM syndrome mouse models [29], so far, allogeneic HSCT remains the only curative option for WHIM syndrome patients. Therefore, based on our experience of HSCT and subsequent short-term follow-up, we believe that HSCT can be considered as a treatment option for WHIM syndrome, including pre-emptive indications in younger patients, before they develop autoimmunity and malignancies at an older age.

## Conclusions

Allogeneic HSCT in WHIM syndrome corrects neutropenia and immunodeficiency, and leads to resolution of autoimmunity and recurrent infections, including warts. However, taking in account potential risks of transplant-related mortality, before larger HSCT experience series with longer follow-up will become available, the decision to perform a HSCT in WHIM syndrome patients must be made individually.

## Data Availability

The dataset used during the current study is available from the corresponding author on reasonable request.
